# A diverse set of solubilized natural fibers drives structure-dependent metabolism and modulation of the human gut microbiota

**DOI:** 10.1128/mbio.00470-25

**Published:** 2025-04-11

**Authors:** Maria X. Maldonado-Gomez, Katharine M. Ng, Riley A. Drexler, Alexandria M. S. Conner, Cory G. Vierra, Nithya Krishnakumar, Hannah M. Gerber, Zachary R. Taylor, Jenna L. Treon, Megan Ellis, Jada K. A. Garcia, James P. Cerney, David G. Chapin, Robnet T. Kerns, Angela M. Marcobal, Steven M. Watkins, Matthew J. Amicucci

**Affiliations:** 1 One Bio Inc., Sacramento, California, USA; University of Maryland School of Medicine, Baltimore, Maryland, USA; Luxembourg Institute of Health, Esch-sur-Alzette, Luxembourg

**Keywords:** gut microbiota, dietary fiber, carbohydrate, human microbiota, short-chain fatty acids, oligosaccharides

## Abstract

Growing evidence suggests that inadequate dietary fiber intake, termed the “fiber gap,” is linked to disease states through disruption of the gut microbiota. Despite this, our understanding of how various fiber structures influence the microbiota and health is limited by the lack of diverse commercially available fibers. Studies have primarily focused on a limited range of fibers, rather than the diverse array of fibers representative of those commonly found in our diets. In this study, we aimed to investigate how naturally derived fibers impact the human microbiota and their metabolic products. We performed a comprehensive structural characterization and functional evaluation of a unique and highly diverse set of new, highly soluble fibers with varied monosaccharide compositions, glycosidic linkages, and polymer lengths. Using an *ex vivo* high-throughput human microbiota platform coupled with metabolomic profiling, we demonstrate that these diverse fibers drive distinct and consistent microbial and metabolic profiles across cohorts of donors in a structure-dependent manner. These metabolic effects were accompanied by both general and donor-specific changes in microbial taxa. Finally, we demonstrate that integrating detailed glycomic characterization with microbial and metabolomic data allowed for prediction of functional outcomes driven by a novel material, pineapple pulp fiber. This work highlights the potential for targeted dietary fiber interventions to modulate the microbiota and improve health outcomes, paving the way for the development of new fiber-rich products with specific health benefits.

**IMPORTANCE:**

Fiber deficiency is associated with numerous disease states, many of which are linked to disruption of the gut microbiota. This study encompasses the first systematic and comprehensive characterization of a diverse collection of naturally derived solubilized fibers and their impacts on the microbiota. The results expand our understanding of the beneficial effects of specific carbohydrate structures naturally found in the human diet, highlighting the potential for designing fiber-based health interventions. The high solubility of these fibers increases both the range of products they can be incorporated in as well as their assayability in experiments, enabling a widespread increase in fiber consumption and positive health impacts.

## INTRODUCTION

Dietary fiber represents one of the most powerful nutritional levers for modulating human health. Many of the mechanisms underlying positive health benefits are mediated by the intestinal microbiota, which ferments fiber and produces important metabolites that modulate metabolic and immune health. The advent of a high-fat, fiber-poor diet across much of the world, termed a “Western diet,” has been accompanied by profound dysbiosis of the microbiota, extinction of key microbial players, and adverse inflammation-related health outcomes, including metabolic disease and autoimmune disorders (1). In light of the clinical evidence supporting the benefits of prebiotic fiber, there has been a push to add fiber back into the diet by enriching processed foods and increasing the usage of fiber supplements. Moreover, increasing interest in prebiotic fibers and gut health is increasing awareness of the “fiber gap,” the population-wide deficiency of fiber in industrialized diets. In fact, due to the increased consumption of refined foods in the USA, adult men and women consume only ~50% of the recommended daily intake of fiber (28–34 g/day for men and 22–28 g/day for women) (2–4). Despite the food industry’s desire to increase fiber in their products, the use of commercially available fibers is problematic in relation to either their lack of formulability, health benefit, or both. These problems stem from the prevalence of synthetic fibers (e.g., dextrinized starches) that meet the definition of dietary fiber but are foreign to the microbiota, and the low solubility of natural fibers that limit their formulation into food products at high enough doses to confer health benefits. Furthermore, from a technical standpoint, the low solubility and high viscosity of many natural fibers hinder researchers’ ability to evaluate their health effects in a systematic, high-throughput manner. Finally, population-wide health benefits have been difficult to demonstrate due to interindividual variability, and commercially available prebiotics have demonstrated contrasting effects on different disease states, challenging the notion of a universal solution for prebiotic supplementation (5–8). All these factors make evident the need to create new naturally derived fibers, but to also understand the impact of their unique structures on a diverse range of microbiomes. As the microbiome community moves toward precision health, it is essential to collectively move past “one size fits all” strategies and to embrace solutions that solve for the interindividual variations within populations.

Numerous human trials support the beneficial health outcomes that result from increased consumption of dietary fiber, including glucose control and lowered cholesterol through microbiota-dependent and -independent mechanisms (9, 10). For example, dietary fiber appears to modulate glucose control through multiple mechanisms, ranging from the production of short-chain fatty acids (SCFAs) by the microbiota to direct inhibition of host dietary enzymes and interference with intestinal glucose absorption (11, 12). Nonetheless, microbiota responsiveness to specific fibers and improvement of health outcomes is considerably variable between individuals (11, 13–17). In numerous studies, effectiveness of dietary fibers in promoting health is correlated with specific microbial drivers not present in all individuals (14, 15, 18). Given the wide variability of the microbiota between individuals across and within different geographic regions (19–22), an approach involving a cohort or population of multiple donors is required to generate robust, generalizable conclusions. However, the limited solubility and gel-forming behavior of many commonly available dietary polysaccharides have restricted the throughput and concentration ranges that can be assayed.

Leveraging the natural diversity of plant fibers is a key strategy to address variability across populations. Currently, commercially available fibers can be broken into three major categories: natural fibers, fructans, and synthetic or dextrinized fibers. Popular natural fibers, including psyllium husk, wheat bran, flaxseed, chia, and oat hull fiber, are produced through mechanical processes like milling and are readily fermented in the gut, as their component polysaccharides have co-evolved with the human microbiota for millennia (23). However, these fibers pose problems for formulation, especially in beverages, due to high intrinsic viscosity and gelling or insolubility. These organoleptic challenges are not only difficult to overcome in food formulations that encourage widespread consumption, but also limit the ability to dose them at high enough levels to promote desired health outcomes. In response to the desire for non-viscous fibers, chicory root inulin and the fructooligosaccharides (FOS) became the preferred fiber source in the functional food and beverage space (24). However, inulin and FOS are known to increase gastrointestinal distress when consumed in amounts far below the dietary fiber recommended dietary intake, which is problematic given the higher doses required to achieve health benefits (13, 25). Recent studies have also demonstrated adverse impacts on allergen-induced type 2 inflammation (5, 26, 27). Finally, while their organoleptic properties are desirable for the food industry, their degradation to fructose during baking and in mildly acidic solutions (<pH 5) results in products with more sugar and less fiber than stated (28). Synthetic (e.g., short-chain FOS, polydextrose) and dextrinized fibers (e.g., soluble corn fiber, wheat dextrin, cassava root fiber, galactooligosaccharides [GOS]) were created to meet the need for highly soluble (hs), non-gelling fiber products with the desired shelf stability (29, 30). The processes used to create synthetic fibers and dextrinized fibers (e.g., enzymatic transglycosylation and pyrodextrinization) result in oligosaccharides with a semi-random distribution of glycosidic linkages—many of which do not exist in nature. The presence of these non-natural structures in dextrinized and synthetic fibers limits fermentability by the microbiota due to a lack of transporters and enzymes for their degradation. The lack of microbially mediated health outcomes has restricted these fibers from meeting the International Scientific Association for Probiotics and Prebiotics (ISAPP) definition of prebiotics, in which providing a health benefit is the third essential component (31). Furthermore, the practice of naming synthetic fibers by the source of the substrate, such as cassava root fiber, only serves to confuse consumers into believing that these fibers are naturally found in the food source from which they are derived.

In this work, we present a novel and diverse set of naturally derived and highly soluble fibers from a wide range of starting plant materials, greatly expanding and characterizing the structural space of functional fibers. We describe a comprehensive high-throughput multidonor platform that couples the structural characterization of these oligosaccharides with a detailed investigation of their functional effects on microbial communities. Previously, we presented the beneficial effects of a highly soluble oat beta-glucan on glucose control (12). This work expands on that knowledge by annotating the relationships between a diverse set of carbohydrate structures and microbial functions across a wide range of donors, and integrating that information in order to construct a framework to successfully predict the impact of a novel collection of fibers on the gut microbiota. The ability to create highly soluble naturally derived fibers paired with a platform for assessing their impacts on the microbiota lays the groundwork for the design of effective prebiotics that can be easily formulated into desirable products with specific health applications.

## RESULTS

### Detailed structural characterization of a diverse set of solubilized natural fibers reveals distinct phyloglycomic groups

**Fig 1:**
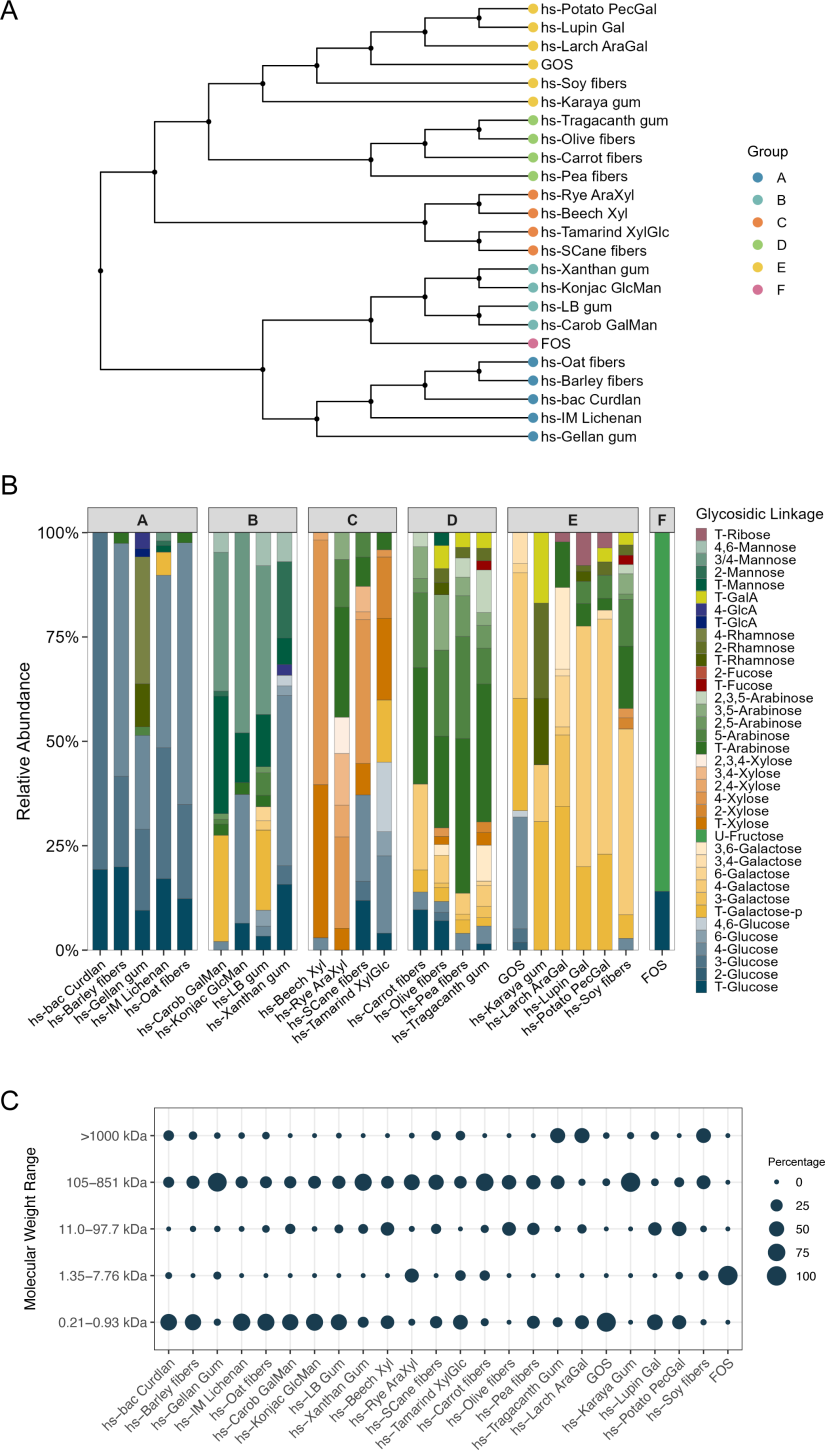
Novel highly soluble fibers represent a diverse set of tool compounds. (**A**) Hierarchical clustering of fibers using Euclidean distance, based on monosaccharide composition of the generated glycan pools. (**B**) Fiber linkage composition, normalized to the monosaccharide composition. Glycan composition groups (A through F) are shown on top of bar plots. (**C**) Size distribution of glycans within each fiber, as determined by size exclusion chromatography with refractive index detection. The area of each circle is proportional to the percentage of the fiber molecules in that corresponding size range.

Fibers from purified polysaccharides as well as fruit and vegetable waste streams were generated using a Fenton chemistry-based depolymerization technology (32, 33). This process created a set of fiber pools with diverse lengths and naturally occurring structures, which were characterized using mass spectrometry to quantify monosaccharide and linkage abundances (Fig. 1). Short, highly soluble fibers made from 22 source materials as well as commercially available GOS and FOS were clustered into groups of similar monosaccharide composition by Euclidean distance, revealing five major clusters, termed phyloglycomic groups (Fig. 1A). Group A was comprised mainly of glucans, with 3- and 4-glucose as the dominant linkages. Group B contained galactomannans and glucomannans, rich in 3/4-linked and terminal mannose, with galactomannans containing mainly terminal galactose and glucomannans 3-linked glucose. Group C contained xylans and arabinoxylans (abundant in 2-xylose), and group D contained mainly arabinans and pectins. Group E contained galactans, which were dominated by terminal and 4-linked galactose. Group F contained FOS, representing fructans (Fig. 1B; Fig. S1A). Notably, the source material taxonomic family did not drive phyloglycomic grouping, highlighting the importance of glycomic characterization of each fiber (Fig. S1B). Groups defined by hierarchical clustering were obtained using monosaccharide relative abundance instead of linkage relative abundance due to the sparsity of linkages, which drove the creation of a large number of phyloglycomic groups containing a single fiber.

Size exclusion chromatography with refractive index detection (SEC-RID) was used to determine the distribution of the oligosaccharide sizes within each of the fibers (Fig. 1C; Fig. S1C). In comparison with the starting polysaccharides, which are too large to be detected using RID methods, the highly soluble fibers contained a range of detectable sizes that were significantly more diverse in size distributions than commercial preparations of FOS and GOS, which contain oligosaccharides in the 0.21 kDa–0.93 kDa and 1.35 kDa–7.76 kDa size ranges, respectively (Fig. 1C). The most abundant molecular size was less than 100 kDa, with the exception of hs-Larch, hs-AraGal, hs-Soy fibers, and hs-Tragacanth gum, where at least 50% of the oligosaccharides were >1,000 kDa. Smaller fiber sizes are likely more readily accessible to microbial fermentation yet still resistant to host digestion.

### Phyloglycomic groups drive differential SCFA production and enrichment of key gut microbiota members

**Fig 2:**
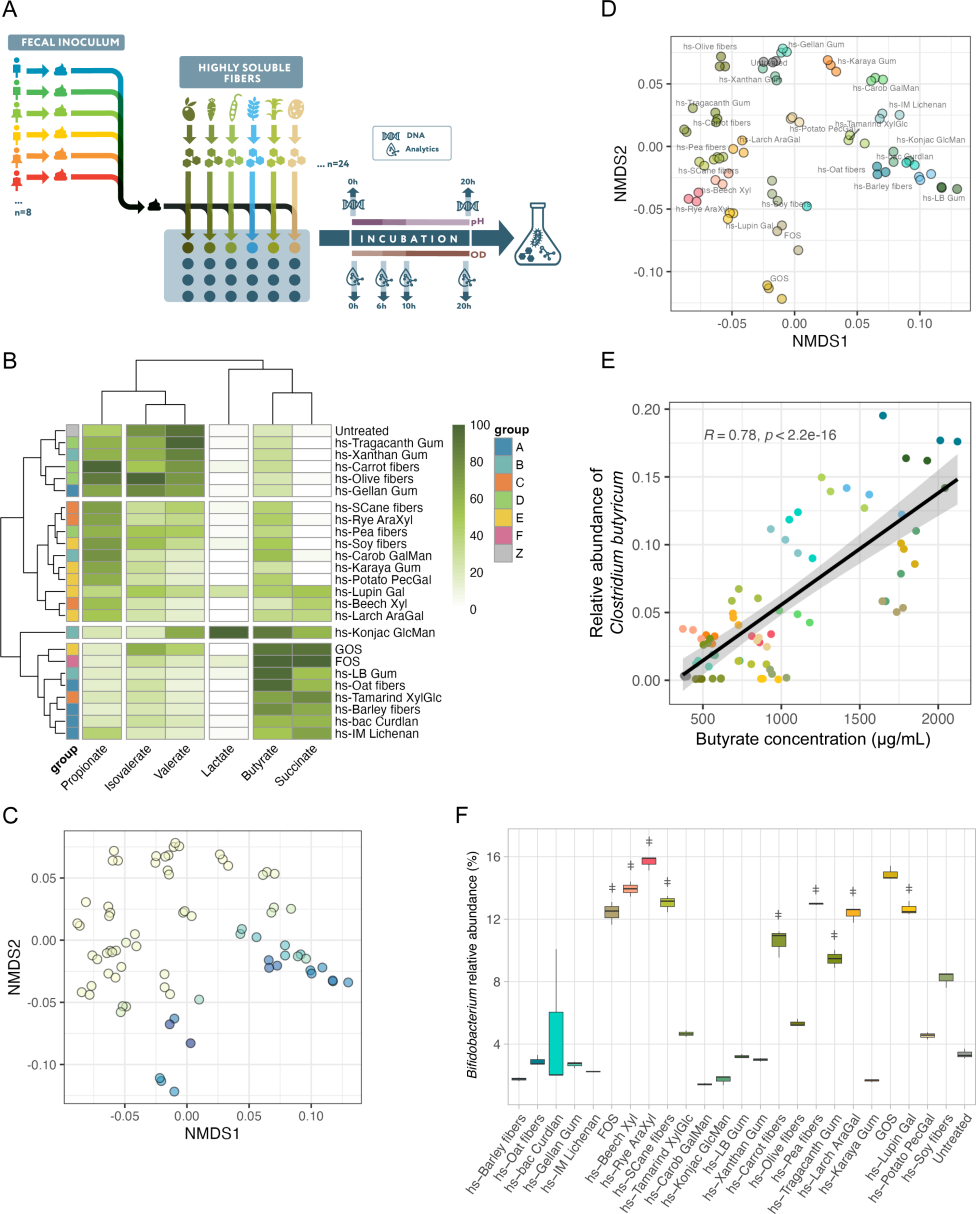
Distinct microbial communities and metabolic profiles result from the interactions between novel highly soluble fibers and fecal microbial communities (A). Experimental design used to test the effects of a novel highly soluble glycan tool set (*n* = 22) as well as GOS and FOS against a single fecal inoculum consisting of a pool of feces from eight healthy donors. Samples were taken throughout the fermentation at 0, 6, 10, and 20 h for SCFA and metabolite quantification. Samples for DNA extraction and microbial community characterization were taken at 0 and 20 h. Untreated controls contained minimal concentrations (0.04%) of di- and oligosaccharides commonly found in the large intestine in order to support basal growth. (B) Distinct SCFA profiles in fecal microbial communities after 20 h of batch fermentation. Mean SCFA levels were scaled by setting the maximum measured value per metabolite to 100%. Samples and SCFAs were grouped using hierarchical clustering based on Euclidean distance. Each glycan is labeled with phyloglycomic tree groups membership to the left of the plot, revealing that SCFA clusters generally matched monosaccharide composition-based phyloglycomic clusters. (C) Non-metric multidimensional scaling (NMDS) clustering of communities derived from pooled fecal inoculum supplemented with different glycans, based on weighted UniFrac. Samples are colored by the butyrate:propionate ratio measured at 20 h of fermentation. (D) NMDS plot of communities in 2C, colored by fiber. Glycans grouped closely to other materials in the same phyloglycomic group. (E) Butyrate concentration (*x*-axis) and relative abundance of *Clostridium butyricum* (*y*-axis) for pooled fecal samples supplemented with different fibers. The production of butyrate was correlated with levels of *C. butyricum* (Pearson correlation coefficient *R* = 0.77). (F) Relative abundance of genus *Bifidobacterium* as determined by 16S rRNA sequencing of fecal fermentations supplemented with fibers revealed a selection of bifidogenic fibers. “ǂ” denotes fibers that are not significantly different from GOS (a known bifidogenic glycan) using the non-parametric Dunn’s test with Benjamini-Hochberg correction.

The detailed glycomic characterization of this unique tool set of highly soluble fibers enabled us to connect structural features with microbial function. In order to characterize the effect of the fibers on the membership and metabolic output of the gut microbiota, we developed a high-throughput platform composed of highly reproducible batch fecal fermentations. The high solubility of the fibers created optically clear solutions (unlike their parent polysaccharides) (Fig. S1D), enabling the continuous measurement of pH, biomass (as a function of OD_600_), and metabolite abundances throughout fermentation and providing the ability to screen dozens of fibers simultaneously. We used a single pooled inoculum (Fig. 2A) consisting of a combination of feces from eight healthy donors (Materials and Methods). Pooling was used to maintain a homogenous and reproducible inoculum and capture functional microbial diversity (34). Batch fecal fermentations were run for 20 h in optimized buffered fermentation media that maintained pH levels in a range relevant to physiological conditions (5.8–6.8).

Fibers differed in their fermentability as assessed by quantifying final biomass and pH (Fig. S2); nevertheless, for all fibers, biomass was higher and final pH was lower than the untreated control. hs-Karaya gum, hs-Tragacanth gum, hs-Carrot fibers, and hs-Gellan gum were the least fermentable, likely partially due to the distribution of oligosaccharides in these fibers skewing toward larger sizes, with the most abundant fractions ranging from 105 to 851 kDa, except for hs-Tragacanth gum (Fig. 1C; Fig. S1C).

As expected, compositionally distinct fibers supported different microbial communities and generated divergent SCFA profiles (Fig. 2B through D; Fig. S3A through E). In general, similar SCFA profiles were derived from fibers sharing similar monosaccharides and linkages (Fig. S3A). For instance, except for hs-Gellan gum (which was poorly fermented based on final OD_600_/pH), the glucans in group A promoted high production of butyrate and succinate (Fig. 2B; Fig. S2B). Xylan-rich group C fibers and galactose-rich group E fibers produced similar levels of both butyrate and propionate, except for hs-Beech Xyl, hs-Tamarind XylGlc, and GOS, which skewed toward butyrate (Fig. 2B and C; Fig. S3E). This can be attributed to the relatively high levels of glucose in GOS and hs-Tamarind XyGlc. Group D, enriched in arabinose-containing glycans, produced a profile dominated by propionate, isovalerate, and valerate (Fig. 2B; Fig. S3C and D).

Microbial communities were monitored after 20 h of fermentation using 16S rRNA sequencing, and compositional changes mirrored observations from SCFA production, as the overall composition of the resulting communities generally clustered by phyloglycomic group (Fig. S3D) and butyrate:propionate ratio, a common metric for metabolism and function of the gut microbiota (Fig. 2C and D). Fiber-supplemented communities that primary produced butyrate were rich in members of *Ruminococcus gnavus* group, *Erysipelatoclostridium*, and *Clostridium sensu stricto 1* (Fig. S4). These included most of the glucans in group A, F (FOS), and GOS. Butyrate concentrations were strongly correlated with levels of *Clostridium butyricum,* a member of *Clostridium sensu stricto 1* (*R* = 0.77, *p* = 4E−16) (Fig. 2E). Thus, in the case of this pooled inoculum, butyrate production was partially attributed to the ability of fibers to support expansion of *C. butyricum*. Other taxa that came up as important at discriminating fiber groups (as determined by random forest analysis) included the genera *Roseburia, Blautia, and Bifidobacterium*, supported by xylose-rich fibers (group C). The majority of galactose-rich fibers (group E) promoted the highest levels of *Bifidobacterium* and supported elevated levels of the butyrate-producing genera *Eubacterium* and *Roseburia*. Finally, communities modulated by arabinose-rich fibers (group D) supported some of the highest relative abundances of *Bifidobacterium* and *Lactonifactor*, *Phascolarctobacterium,* and the family Lachnospiraceae. (Fig. S4).

One of the main nutritional strategies aimed at improving human health through dietary fibers involves harnessing the bifidogenic effect of fibers to enhance overall health, prevent disease, and maintain a robust and balanced gut microbiota. Several novel highly soluble fibers had a strong bifidogenic effect on fecal microbial communities (Fig. 2F), including hs-Beech Xyl, hs-Rye AraXyl, hs-SCane fibers, hs-Carrot fibers, hs-Pea fibers, hs-Tragacanth gum, hs-Larch AraGal, and hs-Lupin Gal. Supplementation with these fibers promoted *Bifidobacterium* levels up to four times higher than the untreated control and non-bifidogenic fibers. Fibers capable of enriching relative levels of *Bifidobacterium* species included preparations rich in galactans, xylans, and arabinose (Fig. 2F). Importantly, the bifidogenic effects of novel highly soluble fibers were equivalent to or better than some of the well-known prebiotics FOS and GOS.

### Fermentation products are driven by fiber structure despite interindividual microbiota variation

**Fig 3:**
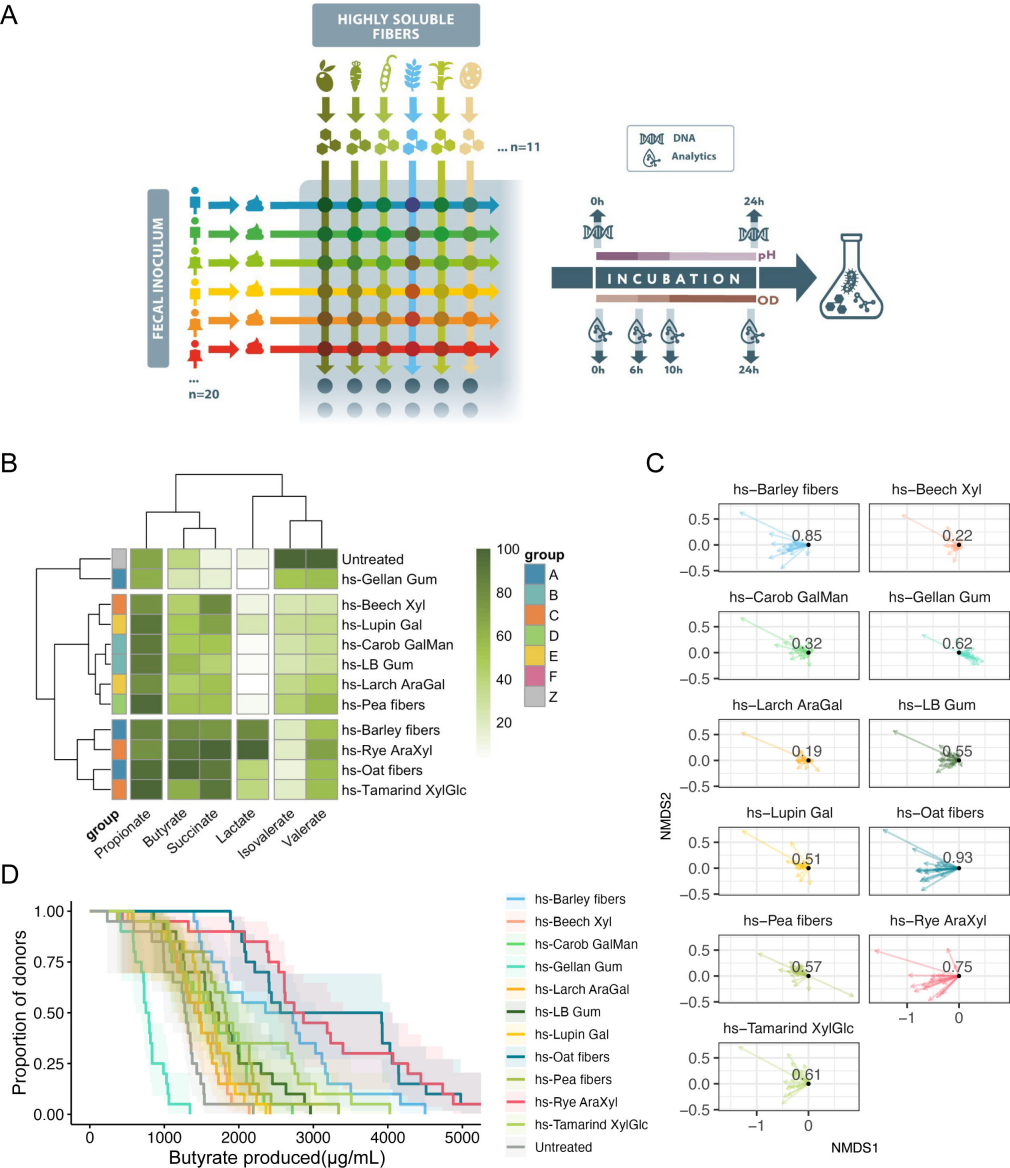
Fibers produce predictable and consistent SCFA profiles across a cohort of human fecal donors (**A**). Experimental design of multidonor platform. Feces from 20 healthy donors (10F, 10M) from Spain were screened against 11 novel highly soluble fibers in 96-well plates using 24 h incubations. Samples were taken throughout the fermentation at 0, 6, 10, and 24 h for SCFA and metabolite quantification. Samples for DNA extraction and microbial community characterization were taken at 0 and 24 h. (**B**) Mean SCFA profiles obtained after 24 h of fermentation. Samples were averaged within treatments and across all donors and scaled to the maximum measured value for each metabolite. Samples and SCFAs were then hierarchically clustered using Euclidean distance, revealing cluster-specific SCFA profiles. (**C**) Relative shifts of SCFA profiles after non-metric multidimensional scaling (NMDS) analysis using Bray-Curtis dissimilarity. Each sample was normalized to the untreated condition for the same donor sample, which was set to 0,0. Cosine similarities were calculated and overlaid on each glycan, demonstrating a high degree of SCFA profile similarity within fibers. (**D**) Complementary cumulative distribution plot of butyrate production after 24 h of fermentation across 20 healthy fecal donor samples, illustrating robust butyrate production by hs-Barley fibers, hs-Oat fibers, and hs-Rye AraXyl across a human cohort.

Previous studies involving dietary fiber have demonstrated heterogeneity in shifts in the microbiota and health responses across individuals (11, 13–15, 35, 36). The high interindividual variability in gut microbiota composition complicates efforts to attribute causality to the glycan-mediated enrichment of a given microbe. Thus, we sought to expand our platform to individually assay a larger number of healthy adult human fecal donors, drawn from a different geographic origin than the initial pooled inoculum (Materials and Methods). These 20 donor stool samples represented a diverse set of microbial communities, with samples containing a range of 13%–30% unique amplicon sequence variants (ASVs) (Fig. S5A). These microbial communities were screened against a subset of the fibers tested in the previous study, focusing on a range of glycans that encompassed the diversity of responses observed with fermentation end products (Fig. 3A). In addition to expanding the set of donors, fecal fermentations were also supplemented with amino acids and bile acids to more accurately simulate the more nutritionally complex intestinal environment.

Our multidonor approach confirmed many of the glycan-driven phenotypes we observed with regard to fermentation end products. Group A, which included hs-Barley fibers and hs-Oat fibers supported robust butyrate production (Fig. 3B). These findings were consistent, not only across donors (Fig. S5 and S6), but also concordant with the behavior of the pooled inoculum (Fig. 2B; Fig. S5B). Across the multidonor cohort, group C, which includes hs-Rye AraXyl, produced higher mean levels of butyrate than in the pooled sample (Fig. S5B and S6). It should be noted that the addition of metabolic precursors in this experiment did cause alterations in some fermentation end products, specifically higher propionate levels, which were likely produced as a result of microbial fermentation of the amino acid precursors (37). Nevertheless, as a whole, SCFAs and other fermentation end products were produced in a similar fashion in the multidonor experiment, suggesting that the effects of the fibers tested can be generalized to a larger population. Importantly, the differences in individual donor SCFA profiles suggest that a multidonor approach is more robust with regard to capturing interindividual variation (Fig. S6).

Changes in SCFA profiles were also evaluated using dimension reduction techniques. Non-metric multidimensional scaling (NMDS) was applied to samples’ SCFA production using the Bray-Curtis dissimilarity metric. hs-Oat fibers, hs-Barley fibers, and hs-Rye AraXyl-supplemented samples clustered more closely with the weighted centroids of the butyrate signal, whereas samples supplemented with hs-Pea fibers shifted more toward propionate (Fig. S5C). Shifts were then calculated relative to each donor’s untreated sample. Cosine similarity scores were calculated as a measure of the similarity in magnitude and directionality of shifts across donors treated with the same glycan. This revealed a high similarity in trajectories within fibers (Fig. 3C); fibers that supported high butyrate (hs-Oat fibers, hs-Barley fibers, and hs-Rye AraXyl) had similar trajectories (and clustered with each other in a k-means clustering analysis) (Fig. S5D and E). Additionally, the high cosine similarity scores of these fiber fractions (0.93, 0.85, and 0.75, respectively; Fig. 3C) suggested that SCFA production supported by these glycans was highly deterministic.

As expected from a diverse donor cohort, treatment with our library of glycan pools resulted in a range of SCFA profiles. Thus, to discern the response to glycan treatment at a multidonor level, we applied a complementary cumulative distribution function approach to metabolite production across our donor cohort (Fig. 3D). This approach enabled us to visualize the percentage of donors that produce a given level of a metabolite, across the range of metabolite concentrations observed, with the percentage of donors dropping off with increasing metabolite concentration. Fibers that supported higher metabolite production across all donors had curves shifted to the right relative to other fibers. This approach revealed that hs-Oat fibers, hs-Rye AraXyl, and hs-Barley fibers supplementation consistently produced the highest butyrate concentrations across our donor cohort, with 50% of the donors capable of producing at least 2,500 µg/mL butyrate, a level which 4 out of 11 of the fibers were unable to support in any donors. Additionally, a higher proportion of donors supplemented with hs-Oat fibers was able to sustain levels of butyrate greater than 4,000 µg/mL, suggesting a higher penetrance of glycan-mediated butyrate production (Fig. 3D).

### Both donor-specific and shared taxonomic shifts drive consistent fiber-dependent SCFA production across a cohort of human microbiotas

**Fig 4:**
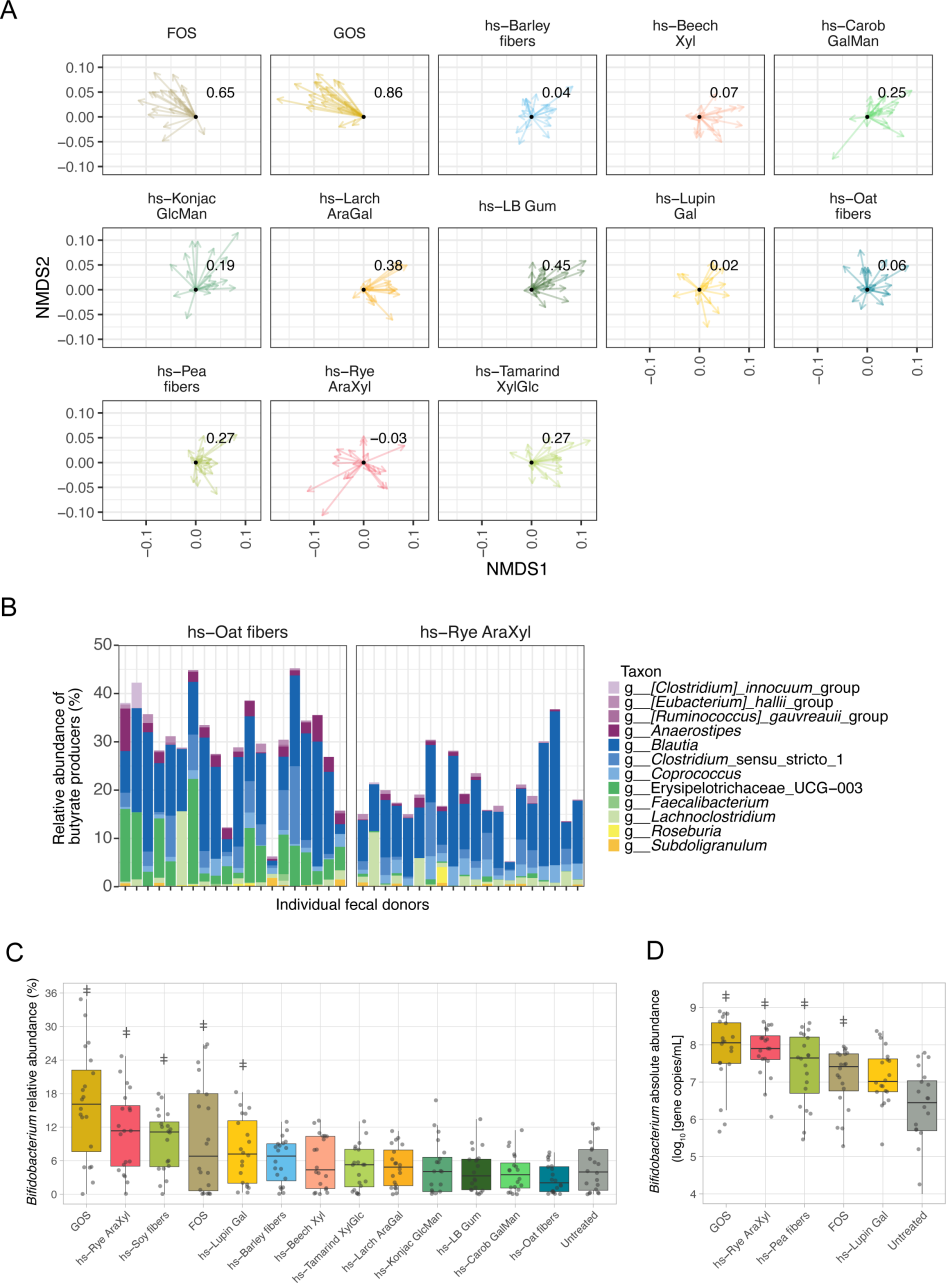
Fiber supplementation results in generalizable and donor-specific effects on microbial membership and microbial metabolism. (**A**) Relative shifts of microbial communities supplemented with fibers, performed after NMDS analysis using weighted UniFrac distances. Each sample was normalized to the untreated condition for the same donor sample, which was set to 0,0. Cosine similarities were calculated and overlaid on each glycan. (**B**) Relative abundance of butyrate-producing taxa reveals distinct taxa were enriched after glycan supplementation with either hs-Oat fibers (left) or hs-Rye AraXyl (right). (C, D*) Bifidobacterium* relative (**C**) and absolute (**D**) abundances revealed three novel highly soluble fibers significantly promoted the abundance of this taxa across a cohort. “ǂ” denotes fibers that are not significantly different from GOS using the non-parametric Dunn’s test with Benjamini-Hochberg correction.

Shifts in SCFAs were accompanied by modulation of the microbial community composition across donors. Samples did not segregate by glycan treatment, suggesting that fiber supplementation likely supported specific individual taxa and did not cause major shifts that overrode the high interindividual variability in starting composition of donors. However, when shifts were normalized relative to starting composition, fibers appeared to cause similar shifts in community composition (Fig. 4A). These shifts were less consistent than the shifts observed in SCFAs as evidenced by their lower cosine similarity scores (Fig. 3C and 4A), suggesting that functional redundancy in the microbiota may underlie similar SCFA profiles, and that the ability of fiber structure to promote functionally redundant taxa may result in deterministic SCFA production. As indicated by the lower similarity scores for community composition than SCFA production (Fig. 3C and 4A), we also observed distinct fiber-responsive taxa in different donors that appeared to be responsible for SCFA production (Fig. 4B). Notably, the taxon responsible for butyrate production in our pooled sample (*Clostridium butyricum*) was not responsible for butyrate production across our new cohort (Fig. 2E). Instead, our analysis revealed both the bloom of donor and glycan-specific butyrate producers.

Supplementation with hs-Oat fibers and hs-Rye AraXyl, substrates that supported robust butyrate production, resulted in distinct profiles of butyrate producers, with some taxa such as *Coprococcus* promoted more by hs-Rye AraXyl, and others including *Blautia*, Erysipelotrichaceae UCG-003, and *[Clostridium] innocuum* group promoted by hs-Oat fibers (Fig. 4B). This suggests that these two fiber preparations enriched different butyrate producers. Additionally, butyrate producers were promoted in a fiber- and donor-specific manner, with *Roseburia* promoted robustly by hs-Rye AraXyl in only one donor, likely due to sparsity in the original donor inocula (Fig. 4B), and pointing to fiber chemistry as the dominant driver for the phenotype.

The bifidogenic effect of highly soluble fibers was also consistently observed for both cohorts and across donors in the multidonor experiment. Of the 13 fibers tested, 5 fibers significantly increased the relative abundance of the genus *Bifidobacterium* (Fig. 4C). This conclusion was substantiated by changes in absolute abundance of this genus as determined by qPCR, showing an increase for hs-Rye AraXyl and hs-Pea fibers that were not significantly different from the well-known bifidogenic prebiotic GOS (Fig. 4D).

### Structural characterization of fibers enables functional prediction of unexplored materials

**Fig 5:**
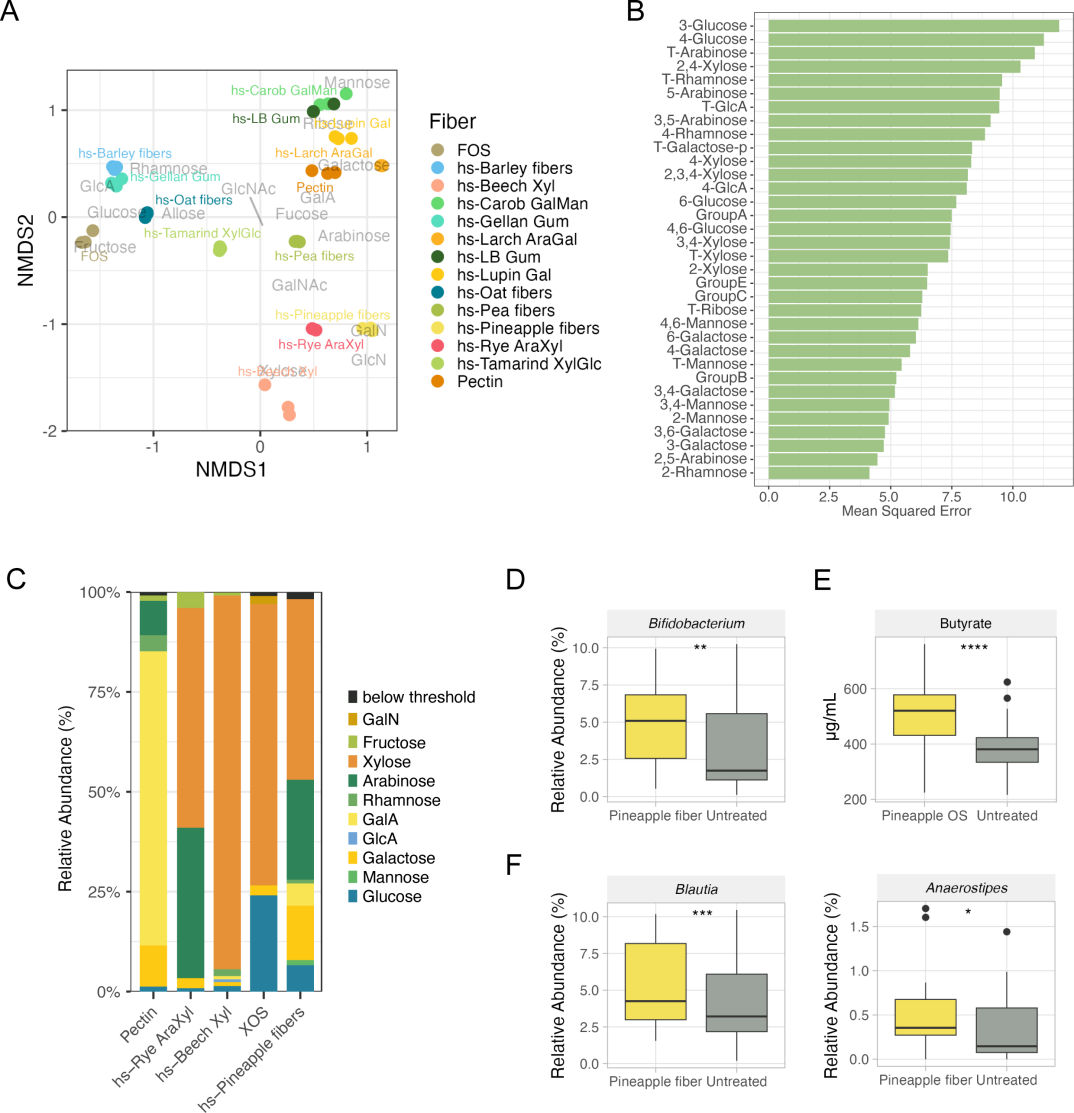
Integrated glycomics, microbial, and metabolomics platform enables prediction of behavior of novel materials (**A**) NMDS analysis performed on fiber linkage composition using Bray-Curtis dissimilarity revealed that hs-Pineapple fiber clustered well with arabinoxylans. (**B**) Random forest regression analysis performed on butyrate production at 24 h revealed carbohydrate structural features important for predicting butyrate levels. (**C**) Monosaccharide abundance of pineapple material compared with other glycomically characterized fibers as reference, including pectin, arabinoxylan, and xylan demonstrating that hs-Pineapple fiber is a predominantly xylan-rich material with a pectin-like component. (**D**) Relative abundance of *Bifidobacterium* after 24 h of fermentation, illustrating the bifidogenic property of hs-Pineapple fiber. (E, F) Quantification of butyrate (**E**) and butyrate producers *Blautia* and *Anaerostipes* (**F**) after 24 h of fermentation, revealing the butyrogenicity of pineapple fiber.

By leveraging the diversity in our oligosaccharides and our multidonor platform, we were able to map a diverse phenotypic landscape against specific carbohydrate features. We hypothesized that our platform’s comprehensive glycomic analysis and existing library of functional results would enable rapid prediction of these health benefits from a new feedstock without the need to optimize soluble fiber production and functional screening. To assess our platform’s prediction capabilities, we performed glycomics analysis on a fiber generated from a different material, pineapple pulp, generated from a side stream created during the process of juice extraction. Previous work on valorizing pineapple pomace and peel waste streams has focused on pectin extraction, although some sources have noted the high hemicellulose content in pineapple stems and peels (38–41). Glycomic analysis revealed that instead of clustering near other pectin-rich materials, pineapple fiber appeared to be more similar to xylan-like materials such as beech xylan and rye arabinoxylan (Fig. 5A). A random forest regression was performed to identify important features in determining butyrate levels, which identified phyloglycomic membership in A and C as well as structural components of both groups (3-glucose, 4-glucose, T-arabinose, and 2,4-xylose among others) (Fig. 5B). A comparison of pineapple fiber composition with pectic and xylan materials demonstrated that pineapple fiber was a hybrid of the two materials (Fig. 5C), with a larger xylan component. Given the strong bifidogenic and butyrogenic effects observed with arabinoxylan in our platform, we predicted that hs-Pineapple fiber would have a similar effect (Fig. 4A). We performed a fecal fermentation supplemented with hs-Pineapple fiber. As predicted, hs-Pineapple fiber increased levels of *Bifidobacterium* species relative to untreated controls (Fig. 5D). We observed that hs-Pineapple fiber also had a butyrogenic effect observed at 24 h (Fig. 5E), which was accompanied by an increase in the relative abundance of butyrate-producing bacterial taxa (Fig. 5F). These results demonstrate that the structure–function relationships elucidated with our diverse set of fibers could be used to predict the functional effects of a novel material.

## DISCUSSION

Dietary fiber exerts multiple beneficial effects on human health, and many of the mechanisms are dependent on their fermentation by the intestinal microbiota. The highly processed modern diet has removed much of the available fiber, leaving a substantial “fiber gap” in consumption that is only partly addressed by the addition of commercial prebiotics. Unfortunately, in addition to a “fiber gap” in terms of fiber consumption, a significant gap also exists with respect to the diversity of dietary fibers consumed and of commercially available prebiotic fibers. Given the close co-evolution of the human microbiota with a plant-based diet for many millennia, this paucity of diversity of naturally derived dietary fiber likely has major implications for the maintenance of beneficial intestinal microbes which are highly susceptible to loss in an environment lacking microbiota-accessible carbohydrates (1).

In this work, we present a diverse set of highly soluble fibers from purified polysaccharides and food products. The solubility of these fiber preparations enables consistent screening at these high concentrations *in vitro*, as well as their incorporation at high, health-relevant concentrations in a variety of foods, both of which present challenges with many bioactive polysaccharides or whole fibers. Additionally, this fiber set expands the explored diversity of currently available highly soluble fibers, which represent a fraction of the diversity of monosaccharides and linkages present in a healthy diet. The depolymerization process used to obtain the soluble fiber preparations enables the upcycling of a variety of feedstocks, including purified polysaccharides as well as inexpensive fruit, vegetable, and grain waste streams, generating a diverse range of naturally derived soluble fibers. The collection of glycans in this study broadens the typical highly-soluble fibers from just three classes (fructans, glucans, and galactans) to include glucomannans, galactomannans, arabinoxylans, arabinans, and pectins. Given that humans and their microbiotas have evolved with all of these glycan classes, studying a more comprehensive set is necessary for a deeper study of microbiota–glycan interactions, especially with regard to primary and secondary degraders and their enzymatic repertoires, which are exquisitely sensitive to different fibers (42).

We leveraged this diverse set of fibers as a set of tool compounds to probe microbiota responsiveness and function across a cohort of healthy human individuals. This platform included glycomic characterization of the fibers as well as high-throughput screening of these fibers against a panel of healthy human fecal donors and the quantification of fermentation end products and changes to microbial composition.

We demonstrated the generalizable and deterministic effects of this set of novel and highly soluble fibers and determined that the production of important fermentation end products such as SCFAs were glycan-driven (Fig. 4A), likely due to the functional redundancy in key fermentative pathways (43). Indeed, although distinct taxa are differentially promoted by beta-glucan and arabinoxylan, the functional redundancy of these taxa appears to underlie the robust butyrate production resulting from supplementation with these fibers. This underscores the concept that the chemical structure of fibers causes a convergence of consistent phenotypes and the importance of generating and characterizing carbohydrate structures upstream of microbiota composition. Functional redundancy and the larger diversity of structures within our fibers likely contribute to the lower degree of between-donor similarity (as assessed by cosine similarity score) of microbial communities supplemented with our fibers relative to commercially available FOS and GOS, which are relatively homogenous (Fig. 4A). Nevertheless, other metabolites that require more specialized pathways such as tryptophan (derived from amino acids) may be produced by specific bacteria that possess unique metabolic repertoires and may thus be highly donor-specific (44). These strains may be enriched by specific dietary fibers, and thus, the discovery of donor-specific metabolic features presents opportunities for probiotic and synbiotic selection. The lower degree of interindividual similarity in microbial composition after supplementation with highly soluble fibers relative to the homogenous effects of FOS/GOS suggests that highly soluble fibers display lower specificity of taxa enrichment. However, it remains to be seen whether prolonged treatment with these fibers results in eventual convergence of microbial communities (45).

In an analysis of a subset of raw materials, we observed that the levels of SCFAs produced by highly soluble fibers and their native polysaccharides are comparable (Fig. S7A). However, in general, the shorter highly soluble fibers exhibited more rapid utilization than their longer starting materials, reaching maximum observed SCFA levels by 10 and 20 h, respectively (Fig. S7B and C). Interestingly, the effects of the novel highly soluble fibers are consistent with metabolic outcomes previously reported for the original natural materials (45). Collectively, this suggests that solubilization primarily facilitates faster metabolism and confirms that the incorporation of these novel highly soluble fibers effectively harnesses natural structures, highlighting the potential for the restoration of synbiotic relationships contributing to human health.

We observed that our fibers were able to support the growth of other health-relevant bacteria such as *Bifidobacterium*. In addition to GOS and FOS, 10 of our fibers promoted *Bifidobacterium* abundances in our pooled screen (Fig. 2F). Of the 13 fibers selected for screening in our multidonor platform, 3 were able to significantly enrich *Bifidobacterium* abundances in the donor cohort (Fig. 4C and D). These results are consistent with the reported plant glycan metabolism of this commensal (46) in *in vivo* and *in vitro* studies (47). Given the high diversity of *Bifidobacterium* species and strains, expanding the range of fibers that can support this important genus is important.

It will be important to conduct future studies to understand how depolymerization of a given material affects trophic networks and downstream health effects. For instance, recent work demonstrated that Fenton depolymerization of beet pulp enhances accessibility for a broader range of *Bifidobacterium* strains (48). Pairing highly soluble fibers (which can be used in high-throughput assays) with their less-soluble native materials thus creates a valuable framework to investigate the relationship between carbohydrate structure, microbial metabolism, and community composition.

Some of the fibers (e.g., hs-Gellan gum) appeared to be less fermentable than others. For these less fermented fibers, longer fermentation times might have captured eventual fermentation and revealed the effect of those fibers on the microbiota and microbial metabolism. These fibers may have been less fermentable because of their larger degree of polymerization (Fig. 1C), or because the microbes capable of degrading them were not present in the donors that we screened. The presence of different fermentability rates and degrees of polymerization within the tested fibers creates the opportunity to combine and create unique preparations that deliver beneficial metabolites throughout the length of the gastrointestinal tract. Additionally, although there appears to be eventual adaptation and tolerance of prebiotic fibers (47), the availability of this diverse set of fibers may facilitate selection of fibers that cause minimal undesirable gastrointestinal symptoms from the beginning of prebiotic supplementation, maximizing consumer satisfaction and adherence.

In this work, soluble fibers were screened against a panel of fecal samples from healthy human subjects, most of which consumed a Mediterranean diet including both vegetables and meat. However, there is a pressing need to screen specific patient cohorts, e.g., infants and people with inflammatory bowel disease (IBD), irritable bowel syndrome (IBS), or celiac disease, many of which are prescribed suboptimal fiber-restricted diets (49). These targeted screens could be coupled with additional readouts (e.g., gas production and effects on gut barrier function and inflammatory markers) tailored to the cohort of interest.

The high-throughput platform in this paper represents a useful tool to connect the structural composition of novel fibers and their effects on human microbes. It is a relatively inexpensive screening tool for selecting the most promising highly soluble fibers, based both on the ability of a fiber to drive a high magnitude response as well as highest consistency across donors before conducting the expensive pre-clinical and clinical testing needed to meet recommended standards (50). As interest grows in valorizing agricultural waste streams, opportunities emerge to generate soluble fibers which need to be screened for potential health benefits. Linking carbohydrate structure with metabolic and taxonomic characterization of a diverse library of fibers enables prediction of the functional effects of novel native materials (Fig. 5). The capacity to generate novel naturally derived and highly soluble fibers and to understand their specific bioactivity will enable the design and production of fibers that can be incorporated into a range of products and used to improve human health on a large scale. This work sets the foundation for future efforts to leverage the structural diversity of dietary fibers as an asset for improving human health.

## MATERIALS AND METHODS

### Production of fibers and glycomic characterization

Fibers were produced by One Bio Inc., from a variety of natural sources through a proprietary modified Fenton chemistry (32, 33). Purified polysaccharides were obtained from Megazyme (Ireland) and Sigma-Aldrich (USA). Commercial glycans FOS and GOS were purchased from Sigma-Aldrich (USA) and Bimuno (Clasado Biosciences, England) correspondingly. Oat, carrot, olive, sugar cane, soy, pea, and pineapple materials were obtained from Purestar Chem (China), Bolthouse Farms (USA), Deoleo Global (Spain), Beneo (Germany), Nutra Food Ingredients (USA), A&B Ingredients (USA), and DSI (Singapore), respectively. Carbohydrates were analyzed for monosaccharide and linkage composition using targeted liquid chromatography (LC)-tandem mass spectrometry (LC-MS/MS) methods. Oligosaccharide size distributions were determined using SEC-RID. Extraction and run details of these methods are provided in Supplementary Methods.

### Oligosaccharide characterization

It is important to note that the number of commercially available linkage standards is limited and does not capture the diversity seen in natural materials. Therefore, glycosidic linkage analysis was performed without a standard curve. The peak area was changed to relative abundance. Furthermore, depending on the degree of branching, glycosidic linkages ionize at different rates. For example, terminal linkages ionize better than branched linkages, resulting in an inflated terminal linkage signal. To account for these ionization differences, the relative abundance of annotated linkages was normalized to monosaccharide abundances. However, the representation of the linkage composition does not include all potential existing linkages in our fibers due to limitations of the analytical technique and available standards, and thus the list of linkages is not fully comprehensive.

### Hydrolyzable monosaccharide analysis

Hydrolyzable monosaccharides were measured in the manner of Amicucci et al., with the following modifications (32). Briefly, polymeric material was hydrolyzed with 4 M trifluoroacetic acid (TFA) for 2 h at 100°C. Liberated monosaccharides were derivatized with PMP (3-methyl-1-phenyl-2-pyrazoline-5-one) and analyzed on an Agilent UHPLC/QqQ mass spectrometer (Agilent Technologies, Santa Clara, CA). Quantitation is performed by employing a standard curve, and peak area is normalized by employing internal standards. Galacturonic acid monosaccharide analysis was measured by enzyme digestion of polymeric material with viscozyme for 1 h at 50°C, then hydrolyzed with 4 M TFA and derivatized with PMP as described above.

### Glycosidic linkage analysis

Glycosidic linkage analysis was performed in the manner of Galermo et al. with modified chromatographic conditions (51, 52). Briefly, polymeric material was permethylated with sodium hydroxide and methyl iodide. Permethylated polymers were hydrolyzed with 4 M TFA for 2 h at 100°C. Liberated monosaccharides were derivatized with PMP and analyzed on an Agilent UHPLC/QqQ mass spectrometer (Agilent Technologies, Santa Clara, CA). Quantitation is reported in units of peak area.

### SEC-RID analysis

Oligosaccharides were analyzed with a 1290 Infinity II LC (Agilent Technologies, Santa Clara, CA) equipped with a size exclusion column (AdvanceBio SEC 130A, 7.8 × 300 mm, 2.5 µm; Agilent Technologies) and 1260 Infinity II RID (Agilent Technologies). Prepared oligosaccharides were diluted to 10 mg/mL in water. LC separation was performed with water (solvent A) and 95% acetonitrile in water (solvent B). A separation gradient was set as follows: 0% B held for 15 min, 0%–30% B in 1 min, return to 0% B in 1 min, and equilibrate at 0% B for 23 min. Peak areas were integrated using Agilent OpenLab CDS Chemstation Edition.

### Fecal fermentation

Static fermentations were conducted in a deep 96-well format, under anaerobic conditions (Anaerobic Chamber Vinyl Type B), using either individual donors or a pool of feces. Fecal inocula were purchased from two different vendors, BioVT (USA) and Microviable Therapeutics (Spain). All fecal donors were healthy, 50% identified as female and the 50% as male, and reported no antibiotic use in the last 3 months. Microviable samples were collected using the GutAlive kit. Anaerobic conditions were maintained during transportation and manipulation to create fecal glycerol stocks. Donors ranged from 30 to 50 years of age and reported consumption of either Mediterranean or Western diets. The pooled fecal inoculum contained samples from donors in the USA, while the multidonor experiment used only the samples collected by Microviable in Spain (see metadata in Tables S2 and S3). Before the start of the experiment, inoculums were activated by mixing 0.2 mL of frozen fecal stocks (at a ratio of 1:3:1 feces, phosphate-buffered saline [PBS], and glycerol) in 4.8 mL of reduced basic fermentation media and incubating for 12 h. Pre-activated inoculum represented a final concentration of 2% of the fermentation reactions. The experiment using pooled inoculum was run by triplicate. Each measurement corresponds to three individual fermentations run during a single experiment. The platform has been validated using multiple biological replicates conducted over several months with a subset of fibers. The fermentation media were optimized to support diverse microbial taxa and control pH within the range of the colon’s physiological conditions and were modified from MacFarlane et al. (53) Importantly, a mix of background sugars (xylan, amylopectin, potato starch, and pectin) was included in the fermentation media at a low concentration (0.04%) to sustain microbial networks and minimize changes due to lack of nutrients which would confound the experimental results. Briefly, base fecal fermentation media was prepared in 75% of the final volume by combining 0.05 g/L bile salts, 1 g/L casein hydrolysate (casitone), 0.67 g/L proteose peptone no. 3, 0.50 g/L cysteine, 0.60 mL/L of 0.1% resazurin in water, 1 mL/L of 500 mg/mL MgSO_4_ * 7H_2_O in water, 10 mL/L hemin solution (1 mg/mL in 0.02 M NaOH), 2 mL/L Tween 80, 3.6 g/L K_2_HPO_4_, 2.3 g/L KH_2_PO_4_, 0.97 g/L NaHCO_3_, 1.17 g/L Na_2_CO_3_, and 9.76 g/L 2-(N-morpholino)ethanesulfonic acid (MES). After filter sterilizing base media, it was combined with 2 mL/L mineral solution, 6 mL/L vitamin solution, 200 mL/L of the 0.2% baseline carbohydrate solution, 0.04 g/L CaCl_2_ · 2H_2_O, and 6 g/L of the highly soluble fiber. Stock solutions of highly soluble fibers were prepared at 2× concentration in water. For the untreated control, fibers were not added. For the multidonor fecal fermentation, the bile salts were replaced with 10 mL/L of bile acid and 40 mL/L of amino acid precursor solutions.

Mineral solution was prepared by diluting in water 0.5 g/L EDTA, 0.2 g/L FeSO_4_·7H_2_O, 0.01 g/L ZnSO_4_·7H_2_O, 0.003 g/L MnCl_2_·7H_2_O, 0.03 g/L boric acid, 0.02 g/L CoCl_2_·6H_2_O, 0.001 g/L CuCl_2_·2H_2_O, 0.002 g/L NiCl_2_·6H_2_O, and 0.003 g/L NaMoO_4_·4H_2_O. The vitamin solution was composed of 0.25 g/L menadione, 0.5 g/L biotin, 0.5 g/L pantothenate, 2.5 g/L nicotinamide (niacin), 0.125 g/L vitamin B12, 1 g/L thiamine, and 1/25 g/L p-aminobenzoic acid dissolved in 63% ethanol. Baseline carbohydrate solution was prepared by combining 0.176 g/L xylan, 0.176 g/L amylopectin, 1.471 g/L potato starch, and 0.176 g/L pectin. This will result in a 20 g/L stock solution. For the bile acid solution, 10 mg/mL sodium glycochenodeoxycholate, 10 mg/mL chenodeoxycholic acid, 10 mg/mL glycocholic acid hydrate, and 10 mg/mL sodium cholate hydrate were dissolved in pure ethanol. Amino acid stock solution consisted of 10 g/L of each of the following amino acids: alanine, arginine, asparagine, aspartic acid, cysteine, cystine, glutamic acid, glutamine, isoleucine, lysine, methionine, phenylalanine, threonine, tryptophan, tyrosine, valine, DL-proline, L-leucine, glycine, L-histidine.

Biomass (absorbance at 600 nm) and pH changes were monitored in real time throughout the fermentation using a fluorescent microplate reader (Synergy H1, Biotek). Fermentation media were supplemented with the fluorescent pH indicator 2′,7′-bis-(2-carboxyethyl)-5-(and-6)-carboxyfluorescein (BCECF), which enabled coupling of pH measurements with optical density (OD) growth measurements as well as measurements at 405 and 475 nm for BCECF signal quantitation. Samples were taken over time (0 h, 6 h, 10 h, and 24 h) to monitor changes in glycan consumption, metabolic profile, and taxonomic membership. Short-chain fatty acids were profiled at all time points, quantifying fermentation end products. In addition, end-of-experiment measurements included 16S rRNA amplicon sequencing and qPCR of microbial communities.

#### DNA extraction and 16S rRNA sequencing

For all studies, DNA was extracted from fecal slurries using the ZymoBIOMICS Kit D4308 and a KingFisher Flex DNA extraction robot. Microbial communities were profiled by sequencing the V4 region of the bacterial 16S rRNA gene amplified using 515F (5′-GTGCCAGCMGCCGCGGTAA-3′) and 806R (5’ GGACTACHVGGGTWTCTAAT-3′) primers.

For the U.S. pooled cohort study, NovaSeq 6000 was used to obtain 250 bp paired-end reads. Novogene USA was contracted to conduct DNA quality control, library prep, and NovaSeq sequencing. For the multidonor study, the University of Minnesota Genomics Center was contracted to conduct library prep and 300 bp paired-end MiSeq sequencing using a dual barcode approach (54).

#### Sequence analysis

Raw demultiplexed reads were processed using QIIME2 2020.11.1 and 2024.2 (55). Briefly, after quality checking, trimming, filtering, and denoising were performed using the “dada2 denoise-paired” plugin in QIIME2. Taxonomic classification of ASVs was performed with the “q2-feature-classifier” plugin and a pre-trained Naive Bayes classifier trained on Silva 138 99% operational taxonomic units (OTUs) from the 515F/806R region of 16S rRNA sequences. Downstream analyses were performed with QIIME2 2024.2. Finally, alpha-diversity and beta-diversity analyses were conducted with QIIME2 plugins and subsequently imported into R for further analysis.

### *Bifidobacterium* absolute quantification by qPCR

Total Bifidobacteria were quantified in fecal fermentation DNA by qPCR using a QuantStudio 3 Real-Time PCR System. PCRs were performed in 20 µL reaction volumes, using PowerTrack SYBR Green Master Mix (10 µL), 20 pmol/µL *Bifidobacterium* genus-specific forward primers (0.5 µL) and reverse primers (0.5 µL), Nuclease-Free Water (8 µL), and DNA template (1 µL). The forward and reverse primers had the following sequences, respectively: 5´–TCG/CGT/CYG/GTG/TGA/AAG–3´ and 5´–CCA/CAT/CCA/GCR/TCC/AC–3´. The PCR conditions consisted of an initial denaturtion phase of 95°C for 2 min, followed by 32 cycles of denaturation at 95°C for 15 s, and an annealing/extending phase for 60 s at 57°C. Absolute quantification was determined from a standard curve prepared using 10-fold serial dilutions of DNA isolated from an overnight broth culture of *Bifidobacterium longum* MiV20-30, which had its colony forming units (CFU) per milliliter quantified through standard plate counting. The detection limit of the qPCR was 4 × 10^4^ CFU/mL, as demonstrated by the standard curve.

### Metabolomics

SCFA analysis was performed as follows. The supernatants were diluted with water (1:20, wt/wt). Twenty microliters of the supernatant dilutions was added to 20 µL of N-(3-dimethylaminopropyl)-N′-ethylcarbodiimide hydrochloride in 5% pyridine. Then 40 µL of 200 mM 2-nitrophenylhydrazine in 80% acetonitrile with 50 mM HCl was added and briefly vortexed prior to incubating for 30 min at 40°C. After incubation, samples were diluted with 400 µL of 10% acetonitrile and centrifuged. Aliquots of the samples were transferred to 96-well plates for UHPLC/QqQ (Agilent Technologies, Santa Clara, CA) analysis.

For the multidonor experiment, an alternative SCFA analysis was performed as follows. Supernatants were diluted with water (1:10, wt/wt), and 20 µL of the diluted supernatants were aliquoted. Next, 20 µL of N-(3-dimethylaminopropyl)-N′-ethylcarbodiimide hydrochloride in 5% pyridine and 40 µL of 200 mM 3-nitrophenylhydrazine in 80% acetonitrile with 50 mM HCl were added and briefly vortexed. Samples were incubated for 30 min at 40°C and subsequently diluted with 400 µL of 10% acetonitrile and centrifuged. Aliquots of the samples were transferred to 96-well plates for UHPLC/QqQ analysis (Agilent Technologies, Santa Clara, CA).

Short-chain fatty acids were analyzed with a 1290 Infinity II LC (Agilent Technologies) equipped with a reverse-phase column (Zorbax Eclipse C18 2.1 × 50 mm; Agilent Technologies) and 6490 Triple Quad LC/MS (Agilent Technologies, Santa Clara, CA). Electrospray ionization mass spectrometry (ESI-MS) conditions were performed in positive mode (negative mode for the multidonor experiment), and the dynamic multiple reaction monitoring mode was used to monitor the precursor and product ion transitions. Peak areas were quantitated using Agilent Quantitative Analysis software, and areas were normalized to internal standards and compared to an external standard curve for quantitation.

Conclusions were drawn using the 24 h time point since, for the most part, SCFAs accumulated with the progression of the fermentation. An exception was lactate, which was produced early in the fermentation and subsequently transformed into other metabolites. Analysis of all the sampled time points revealed that lactate production peaked at 10 h. Lactate production is also glycan dependent, with hs-Pea fibers being the top lactate producer (Fig. S5F). Sampling throughout the fermentation allowed us to explore reaction dynamics. However, results presented in this publication focused on levels observed at 24 h.

### Statistics

All statistical analyses were performed using R version 4.2.0 (56, 57).

Fibers were clustered based on monosaccharide composition using hierarchical cluster analysis on dissimilarities determined by Euclidean distance.

NMDS was used to condense and visualize microbial taxonomical community membership and SCFA profiles. Clustering of microbial communities is based on weighted UniFrac distances which were calculated using QIIME2 tools. Gower’s distance was used to determine SCFA profiles’ dissimilarity. Adonis permutational analysis of variance (PERMANOVA; adonis in vegan v.2.6-6) was used to determine significant differences between groups. *P*-values are included in the plots when appropriate.

Normalization of multidonor SCFA and 16S NMDS ordination was performed in R for each donor by first shifting the coordinates of the untreated sample to (0,0) and transforming the remaining samples by the same shift. The displacement of each fiber treatment relative to untreated was then calculated. The mean cosine similarity score for a given glycan was calculated by first calculating the pairwise cosine similarity scores between each donor and every other donor, and then averaging all pairwise cosine similarity scores.

Heatmaps were generated using pheatmap, a function implemented in R with the package pheatmap (v.1.0.12). Mean SCFA levels were scaled by setting the maximum measured value per metabolite to 100%, and fibers and SCFAs were clustered using hierarchical clustering based on Euclidean distance, which are the default methods in pheatmap.

Differences in the absolute and relative abundance of genus *Bifidobacterium* were determined by using the non-parametric Dunn’s test with Benjamini-Hochberg correction, where GOS was the reference group. In figures, “ǂ” denotes fibers that are not significantly different from GOS. Kruskal-Wallis non-parametric analysis of variance (ANOVA) followed by Dunn’s test with Benjamini-Hochberg was used to evaluate differences in the relative abundance of the phyloglycomic groups’ discriminating taxa.

To determine what taxonomic groups are capable of discriminating between phyloglycomic groups as well as features capable of predicting butyrate production, random forest analysis was used with the randomForest function implemented in R (v4.7-1.1) (58). The number of trees to grow was set to (ntree = 500) and the number of variables randomly sampled as candidates at each split was 5 (mtry = 5).

To create complementary cumulative distribution curves, survival functions (a subset of complementary cumulative distributions) were computed in R. Instead of calculating the proportion of donors surviving at a given time, the proportion of donors able to produce a given butyrate level at 24 h was calculated. Surv and survfit from the survival package were used to compute survival curves, and then the ggsurvplot function from the survminer package was utilized to plot the resulting survival curves.

## Data Availability

Sequence data for 16S rRNA sequences, mass spectrometry files, and qPCR data were deposited on Figshare, under DOI 10.6084/m9.figshare.27478197.

## SUPPLEMENTAL MATERIAL

Supplemental figures mbio.00470-25-s0001.pdf Figures S1-S7 and captions for Tables S1-S3.

Supplemental tables mbio.00470-25-s0002.xlsx Tables S1-S3.

ASM does not own the copyrights to Supplemental Material that may be linked to, or accessed through, an article. The authors have granted ASM a non-exclusive, world-wide license to publish the Supplemental Material files. Please contact the corresponding author directly for reuse.
