# Supplementary material for: A diverse set of solubilized natural fibers drives structure-dependent metabolism and modulation of the human gut microbiota
Source: mBio. 2025 Apr 11;16(5):e00470-25. doi: 10.1128/mbio.00470-25 (PMC12077125; doi:10.1128/mbio.00470-25)
Supplement: Supplemental figures — Figures S1-S7 and captions for Tables S1-S3. [file mbio.00470-25-s0001.pdf]

Supplemental Material

A Diverse Set of Solubilized Natural Fibers Drives Structure-Dependent Metabolism and Modulation of the Human Gut Microbiota

Maria X. Maldonado-Gomez*, Katharine M. Ng*, Riley A. Drexler, Alexandria M. S. Conner, Cory G. Vierra, Nithya Krishnakumar, Hannah M. Gerber, Zachary R. Taylor, Jenna L. Treon, Megan Ellis, Jada K. A. Garcia, James P. Cerney, David G. Chapin, Robnet T. Kerns, Angela M. Marcobal,Steven M. Watkins, Matthew J. Amicucci

Figure S1: Fiber monosaccharide composition and size distributions
A) Glycan monosaccharide composition expressed in relative abundance. Glycan composition groups (A-F) are shown on top of barplots. **B)** Hierarchical clustering of fibers using Euclidean distance, based on monosaccharide composition of the generated oligosaccharides pools, labeled with taxonomic family of source material. **C)** Heatmap of the glycan size distributions within novel highly soluble fibers and commercially available FOS and GOS. Shade of heatmap represents the percentage of glycans within a fiber in a given size range. **D)** Photos of solutions of highly soluble fibers at a concentration of 40 mg/ml in water on the top row demonstrate clarity and solubility of Fenton depolymerized fibers. Bottom row contains photographs of starting oat polysaccharide material, partially purified oat polysaccharide during processing to generate depolymerized fiber, as well as the resulting highly soluble fiber end product, each at a concentration of 12 mg/ml in water.

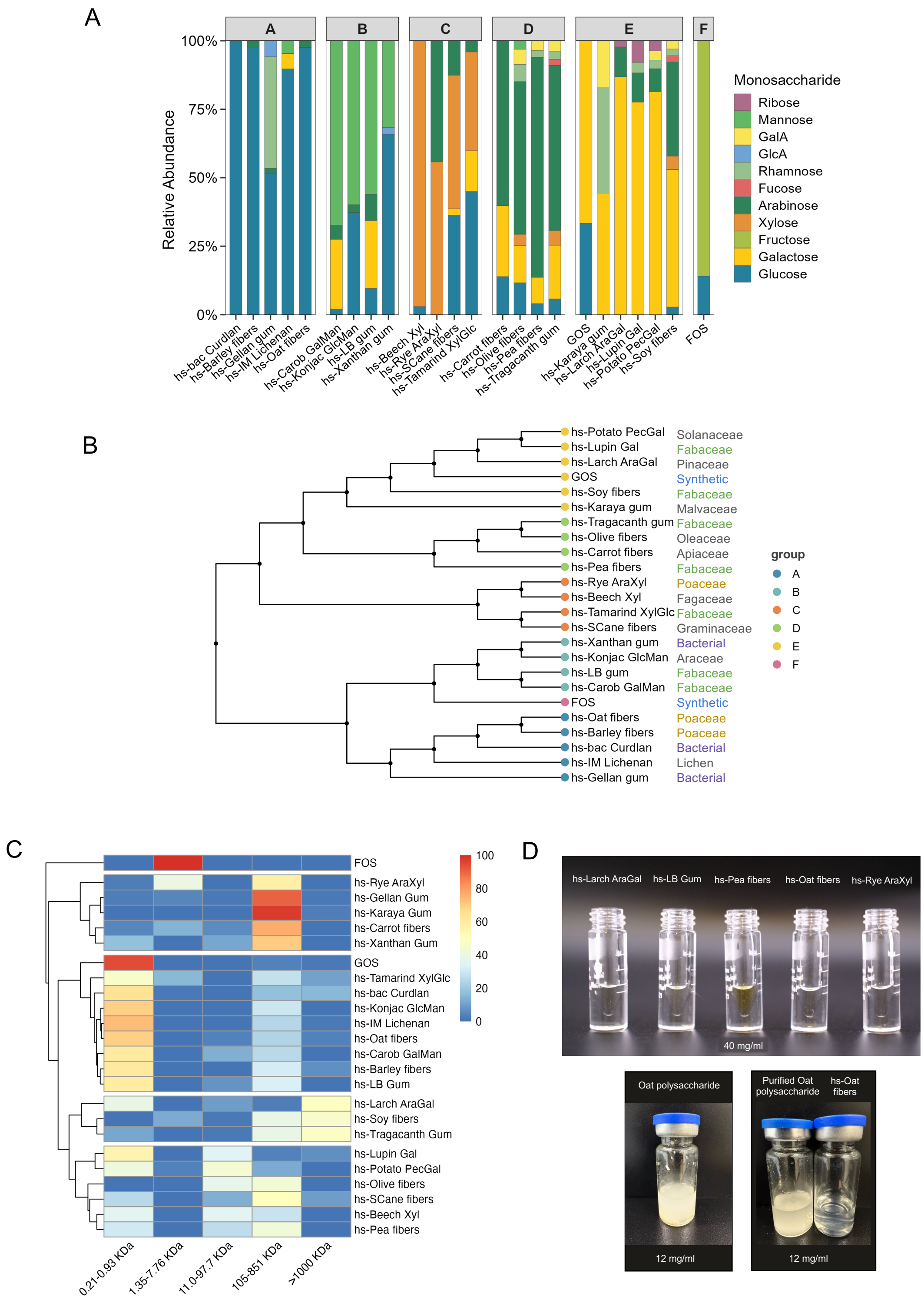

Figure S2: OD600, pH and butyrate:propionate ratio of pooled fecal inoculum fermentations supplemented with individual fibers. A) OD600 of fecal fermentations involving the pooled fecal inoculum supplemented with fibers. **B)** Final pH of fiber-supplemented fecal fermentations. **C)** Final butyrate:propionate ratios of cultures supplemented with fibers, separated by phyloglycomic group.

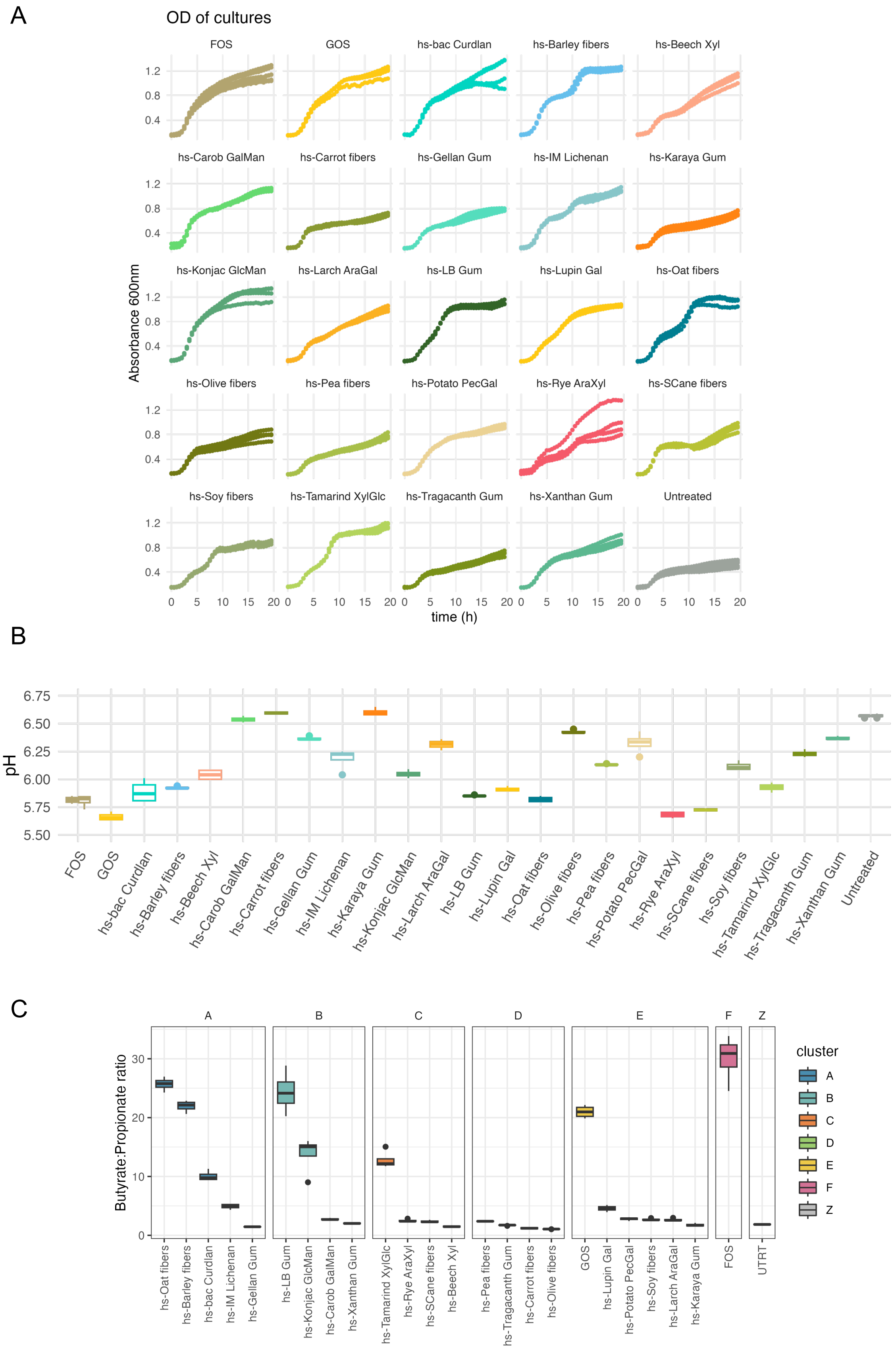

Figure S3: SCFA profiles organized by phyloglycomic group and NMDS plots of SCFA profiles (gower) and microbial composition (Weighted UniFrac) colored by butyrate, propionate and/or phyloglycomic group.

A) Distinct SCFA profiles in fecal microbial communities after 20 hours of batch fermentation, clustered by phyloglycomic group. As in Figure 2B, mean SCFA levels were scaled by setting the maximum measured value per metabolite, to 100%. SCFAs were grouped using hierarchical clustering based on Euclidean distance. Each glycan is labeled with phyloglycomic tree groups membership to the left of the plot, and rows are grouped by phyloglycomic group. **B)** Non-metric multidimensional scaling (NMDS) of SCFA profiles derived from pooled fecal samples supplemented with different glycans, based on Gower distance, colored by phyloglycomic group. **C)** Non-metric multidimensional scaling (NMDS) of communities derived from pooled fecal samples supplemented with different fibers, based on weighted UniFrac. Points are colored by butyrate production at 20h. **D)** Non-metric multidimensional scaling (NMDS) of communities derived from pooled fecal samples supplemented with different fibers, based on weighted UniFrac. Points are colored by propionate production at 20h. **E)** Non-metric multidimensional scaling (NMDS) of communities derived from pooled fecal samples supplemented with different fibers, based on weighted UniFrac. Points are colored by phyloglycomic group. **F)** Butyrate:propionate ratio at 20h produced by cultures supplemented with different fibers.

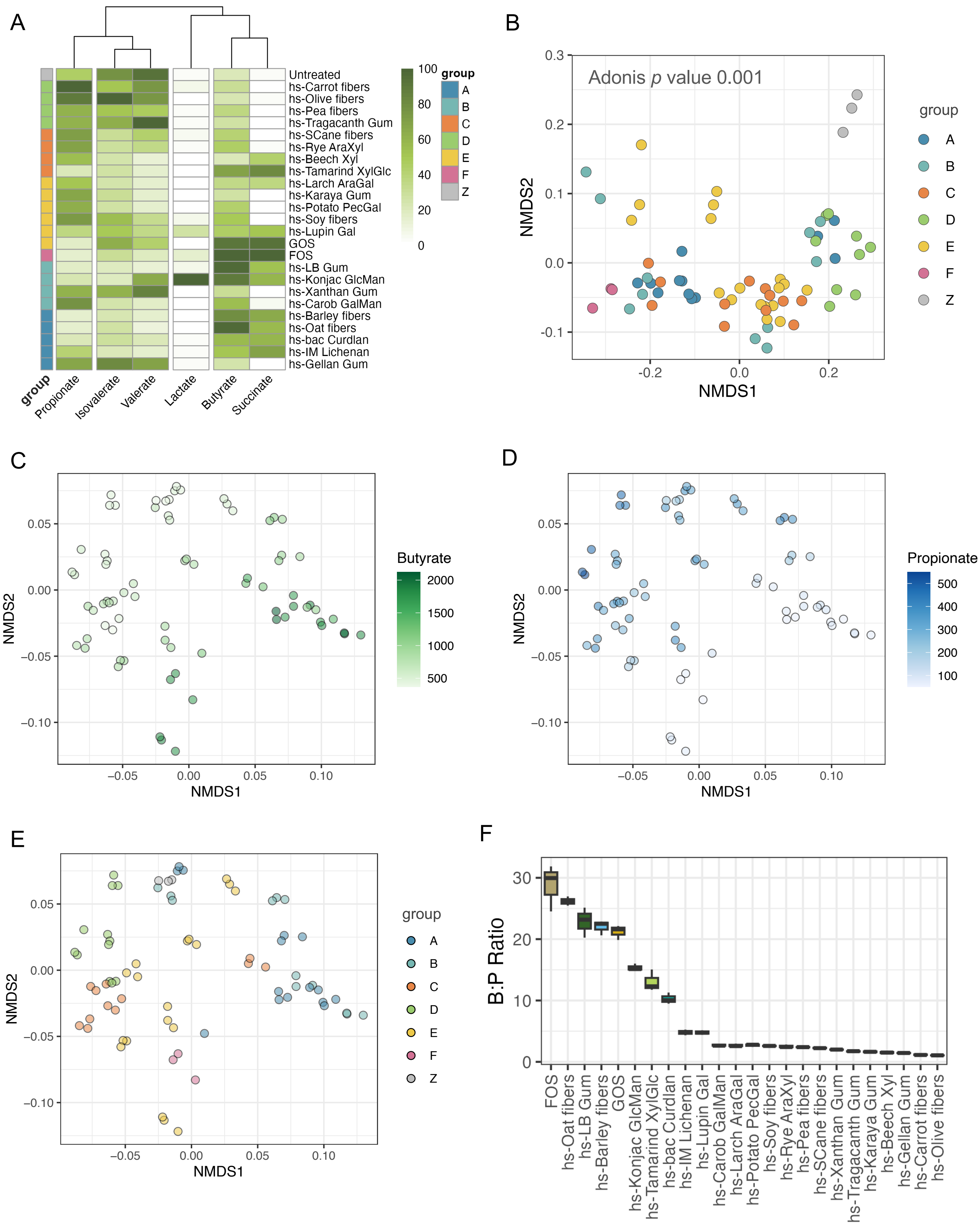
A
B
Untreated
hs-Carrot fibers
hs-Olive fibers
hs-Pea fibers
hs-Tragacanth Gum
hs-SCane fibers
hs-Rye AraXyl
hs-Beech Xyl
hs-Tamarind XylGlc
hs-Larch AraGal
hs-Karaya Gum
hs-Potato PecGal
hs-Soy fibers
hs-Lupin Gal
GOS
FOS
hs-LB Gum
hs-Konjac GlcMan
hs-Xanthan Gum
hs-Carob GalMan
hs-Barley fibers
hs-Oat fibers
hs-bac Curdlan
hs-IM Lichenan
hs-Gellan Gum
group
Propionate
Isovalerate
Valerate
Lactate
Butyrate
Succinate
Adonis p value 0.001
NMDS1
NMDS2
C
D
E
F
Butyrate
Propionate
B:P Ratio

Figure S4: Phyloglycomic group-discriminating bacterial taxa. A) Importance of taxa capable of discriminating microbial fecal communities between fiber groups as determined by random forest classifier analysis. **B)** Scaled relative abundance of taxa identified by random forest analysis as important in discriminating phyloglycomic groups represented in a heatmap. **C)** Relative abundance of a subset of discriminating taxa in cultures supplemented with highly soluble fibers, expressed as a fraction. Boxplots are separated and colored by phyloglycomic group. Differences were established by Kruskal-Wallis non-parametric ANOVA, followed by Dunn's Test with Benjamini-Hochberg correction.

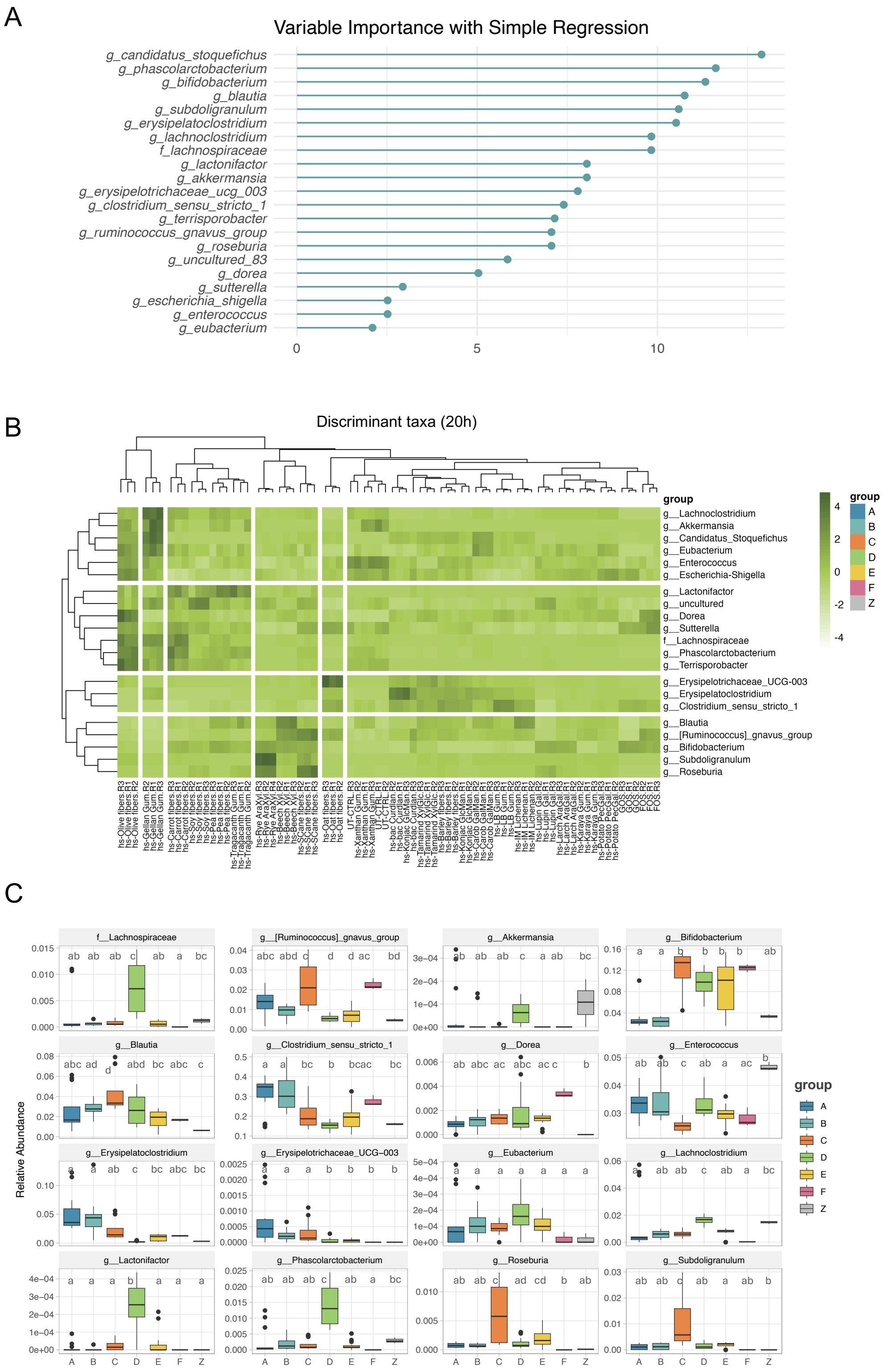

Figure S5: Diverse microbial communities in a multidonor platform produce consistent SCFA profiles when supplemented with fibers. **A)** Shared (green) and unique (yellow) ASVs in donor samples used in the multidonor experiment reveal a high number of unique ASVs in individual donors. **B)** Comparison of mean SCFA profiles between the pooled and multidonor experiments demonstrate a general concordance between the pooled and multidonor experiment. Heatmaps were filtered to include the subset of glycans investigated in the multidonor experiment. **C)** Non-metric multidimensional scaling (NMDS) of communities derived from the multidonor fecal samples supplemented with different glycans, based on Bray-Curtis dissimilarity of SCFA profiles. Centroid of each SCFA is overlaid on the plot. **D)** Vectors representing the mean relative shift of SCFA profiles of individual donor samples supplemented with fibers demonstrate distinct trajectories for high butyrate-supporting fibers. **E)** *k*-means clustering of mean vectors represented in S5D. **F)** Quantification of lactate 10h after start of fermentations supplemented with highly soluble fibers.

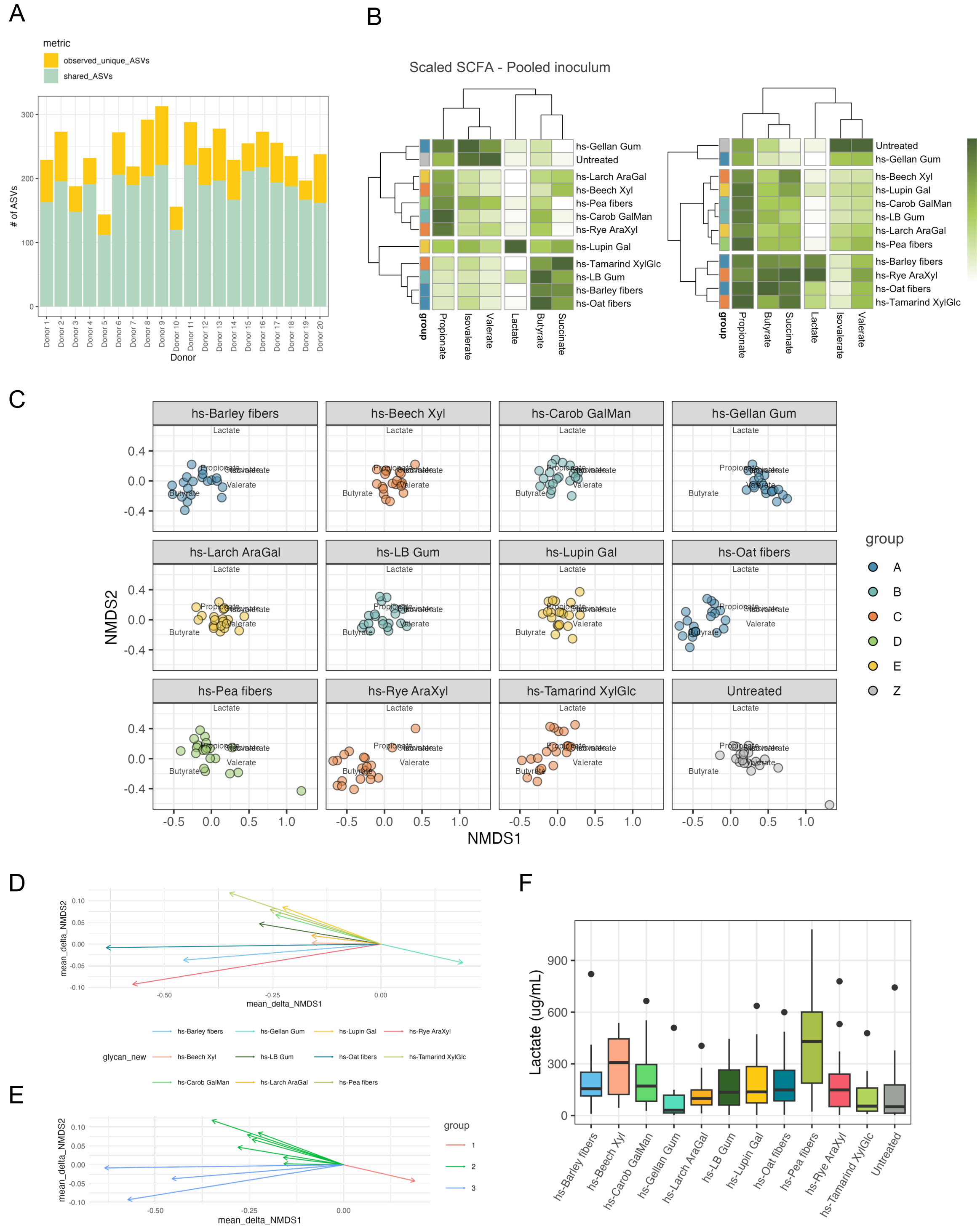

Figure S6: Individual donor SCFA profiles at 24h after fiber supplementation. SCFA profiles for individual donors obtained after 24h of fermentation with fibers. Samples were scaled to the maximum measured value for each metabolite by donor.

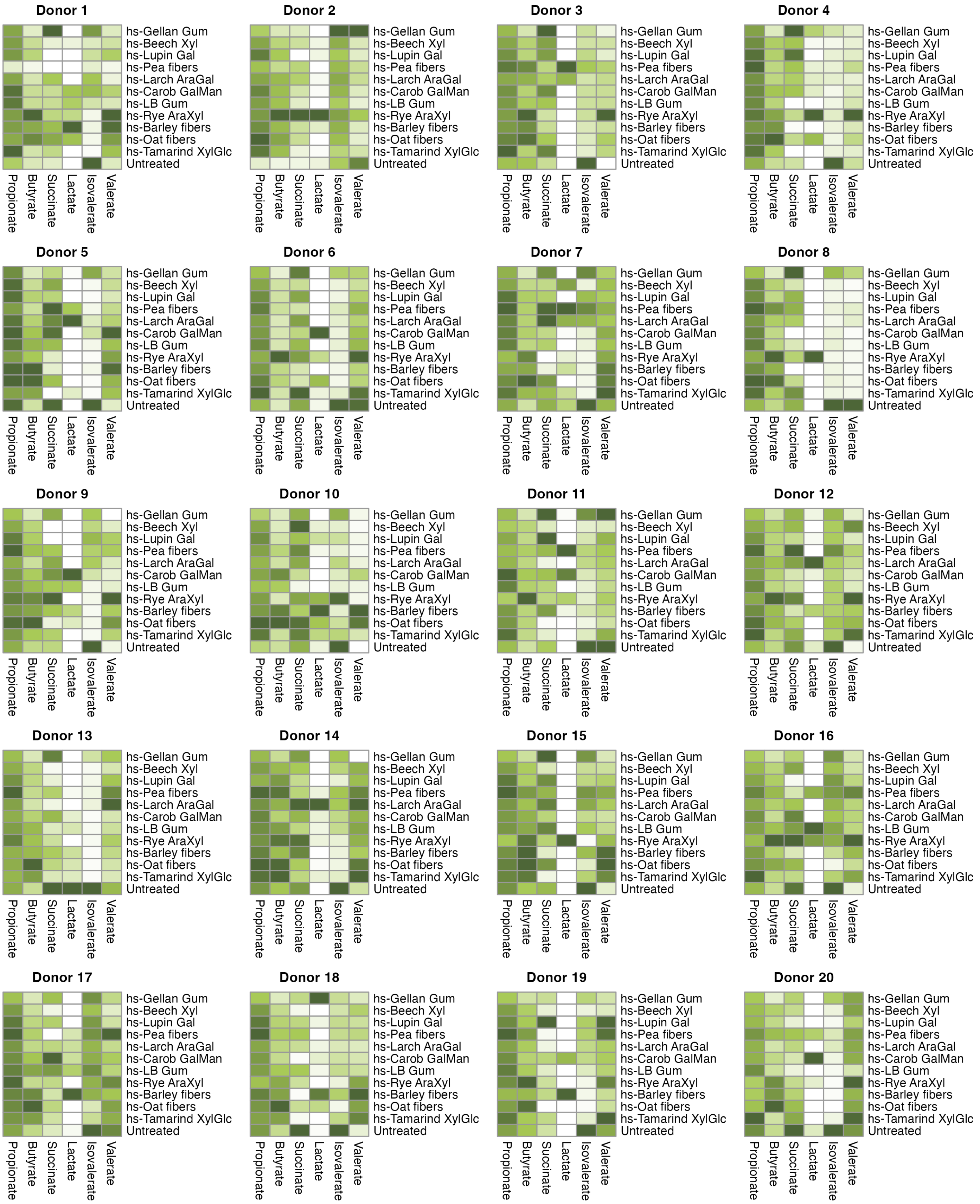

Figure S7: Comparison of SCFA production between a subset of highly soluble fibers and their polysaccharide starting materials. **A)** Overall levels of SCFAs produced by highly soluble fibers and their native polysaccharides are comparable. Plot of the sum of measured SCFAs after 20h of fermentation supplemented with either highly soluble fibers (open circles) or their starting polysaccharide materials (filled circles). **B**) and **C)** Sum of measured SCFAs produced by fermentations at 10 hours (grey, open diamonds) and 20 hours (black, filled circles), supplemented with (**B**) starting polysaccharide materials or (**C**) highly soluble fibers.

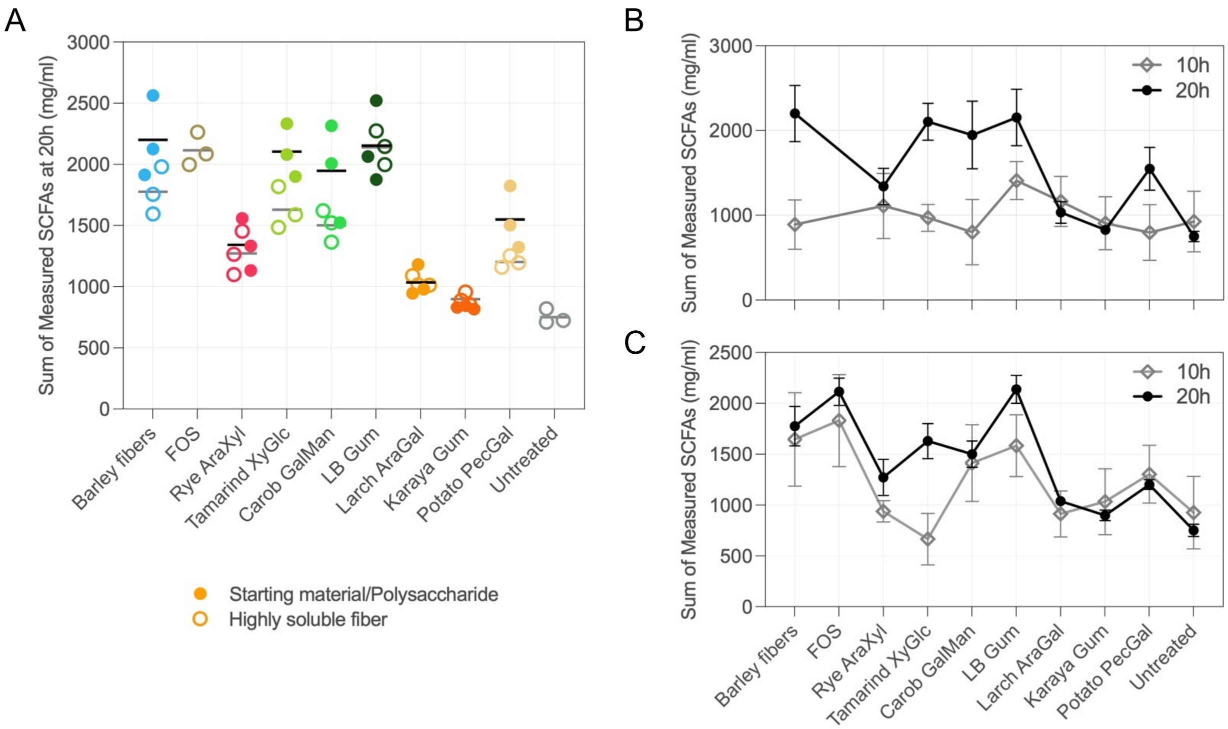

Supplementary Table Legends

Supplementary Table 1: Table of highly soluble fibers and sources for starting materials.

Supplementary Table 2: Subject metadata for US fecal donors in pooled experiment.

Supplementary Table 3: Subject metadata for Spanish fecal donors in multidonor experiment.
